# Case Definitions Used During the First 6 Months of the 10th Ebola Virus Disease Outbreak in the Democratic Republic of the Congo — Four Neighboring Countries, August 2018–February 2019

**DOI:** 10.15585/mmwr.mm6901a4

**Published:** 2020-01-10

**Authors:** Alexandra M. Medley, Oscar Mavila, Issa Makumbi, Felicien Nizeyemana, Angela Umutoni, Hélène Balisanga, Yona Kenyi Manoah, Aimee Geissler, Sudhir Bunga, Gene MacDonald, Jaco Homsy, Joseph Ojwang, Raimi Ewetola, Pratima L. Raghunathan, Amanda MacGurn, Kimberly Singler, Sarah Ward, Shahrokh Roohi, Vance Brown, Trevor Shoemaker, Richard Lako, Adeline Kabeja, Allan Muruta, Leopold Lubula, Rebecca Merrill

**Affiliations:** ^1^Division of Global Migration and Quarantine, National Center for Emerging and Zoonotic Infectious Diseases, CDC; ^2^Epidemic Intelligence Service, CDC, ^3^Programme National d’hygiène aux Frontières, Democratic Republic of the Congo; ^4^Uganda Ministry of Health; ^5^Rwanda Biomedical Center; ^6^South Sudan Ministry of Health; ^7^Division of Foodborne, Waterborne, and Environmental Diseases, National Center for Emerging and Zoonotic Infectious Diseases, CDC; ^8^Division of Global HIV & TB, Center for Global Health, CDC; ^9^Division of Global Health Protection, Center for Global Health, CDC; ^10^Office of the Director, Center for Global Health, CDC; ^11^Division of High-Consequence Pathogens, National Center for Emerging and Zoonotic Infectious Diseases, CDC; ^12^Division de Lutte contre la Maladie, Democratic Republic of the Congo.

On August 1, 2018, the Democratic Republic of the Congo (DRC) declared its 10th Ebola virus disease (Ebola) outbreak in an area with a high volume of cross-border population movement to and from neighboring countries. The World Health Organization (WHO) designated Rwanda, South Sudan, and Uganda as the highest priority countries for Ebola preparedness because of the high risk for cross-border spread from DRC (*1*). Countries might base their disease case definitions on global standards; however, historical context and perceived risk often affect why countries modify and adapt definitions over time, moving toward or away from regional harmonization. Discordance in case definitions among countries might reduce the effectiveness of cross-border initiatives during outbreaks with high risk for regional spread. CDC worked with the ministries of health (MOHs) in DRC, Rwanda, South Sudan, and Uganda to collect MOH-approved Ebola case definitions used during the first 6 months of the outbreak to assess concordance (i.e., commonality in category case definitions) among countries. Changes in MOH-approved Ebola case definitions were analyzed, referencing the WHO standard case definition, and concordance among the four countries for Ebola case categories (i.e., community alert, suspected, probable, confirmed, and case contact) was assessed at three dates (*2*). The number of country-level revisions ranged from two to four, with all countries revising Ebola definitions by February 2019 after a December 2018 peak in incidence in DRC. Case definition complexity increased over time; all countries included more criteria per category than the WHO standard definition did, except for the “case contact” and “confirmed” categories. Low case definition concordance and lack of awareness of regional differences by national-level health officials could reduce effectiveness of cross-border communication and collaboration. Working toward regional harmonization or considering systematic approaches to addressing country-level differences might increase efficiency in cross-border information sharing.

Ebola case definitions provided by the MOHs in DRC, Rwanda, South Sudan, and Uganda were compared during the first 6 months of the DRC outbreak. Because Rwanda, South Sudan, and Uganda had no reported cases at the time, their case definitions were for the preparedness phase of emergency response. Three dates for comparison were chosen to assess definitions: the start of the DRC outbreak (August 1, 2018), the period before the peak (November 15, 2018), and 6 months into the outbreak (February 1, 2019).

Criteria for five Ebola case definition categories (community alert, suspected, probable, confirmed, and case contact) were reviewed, accommodating minor wording differences. For example, a confirmed case category might have had three criteria: “suspected case, laboratory-confirmed by reverse transcription–polymerase chain reaction (RT-PCR),” “suspected case, laboratory-confirmed by IgM (immunoglobulin M) antibody presence,” or “suspected case with a positive laboratory test.” Alerts at points of entry (where travelers were screened for Ebola) were considered an additional category; point of entry alerts were either an independent category or described within the “community alert” category.

The number of criteria present for each country was divided by the total number of possible criteria listed by all of the four countries to calculate the percentage of criteria present per category. The category percentage concordance (overlap) across the four countries was calculated by dividing the number of criteria used by all countries by the total number of possible criteria for that category. Countries that did not use a category were excluded from that category’s analysis. Rwanda, South Sudan, and Uganda, the three countries bordering DRC, reported changing their Ebola case definitions in response to the context and perceived risk for an outbreak, especially during the early months of the DRC outbreak. During the 6 months from August 1, 2018, through February 1, 2019, each country revised its case definitions two to four times. The interval between revisions varied from 1 month to 5 months. All four countries revised their definitions in January 2019 after DRC’s Ebola incidence peaked in December 2018. Uganda did not include the probable category throughout the 6 months, and South Sudan removed that category by November 2018 (Table 1). Rwanda’s case definition did not include a community alert category until January 2019. Only Uganda included the case contact category consistently throughout the 6 months, although other countries defined case contact at one of the three dates or might have listed criteria in other surveillance documents.

**TABLE 1:** Number of criteria per category for all major Ebola virus disease (Ebola) case definition categories at three dates during the first 6 months of the 10th Ebola outbreak in the Democratic Republic of the Congo (DRC), with the World Health Organization (WHO) standard Ebola case definition for reference — DRC, Rwanda, South Sudan, and Uganda, August 2018–February 2019

Date/Country	Case definition category					
	No. (%) suspected	No. (%) probable	No. (%) confirmed	No. (%) community alert	No. (%) case contact	No. (%) for POE
**August 1, 2018**						
**Total no. of criteria** **used by all countries**	**12**	**3**	**5**	**9**	**14**	**1**
DRC	6 (50)	3 (100)	2 (40)	3 (33)	8 (57)	1 (100)
Rwanda	6 (50)	1 (33)	2 (40)	None*	None	None
South Sudan	3 (25)	2 (67)	2 (40)	4 (44)	5 (36)	None
Uganda	6 (50)	None	2 (40)	4 (44)	7 (50)	None
WHO	4 (33)	1 (33)	3 (60)	3 (33)	9 (64)	None
**November 15, 2018**						
**Total no. of criteria** **used by all countries**	**13**	**3**	**6**	**8**	**12**	**2**
DRC	6 (46)	2 (67)	1 (17)	3 (34)	None	None
Rwanda	6 (46)	1 (33)	2 (33)	None	None	None
South Sudan	7 (54)	None	2 (33)	6 (75)	None	2 (100)
Uganda	6 (46)	None	2 (33)	3 (34)	7 (58)	None
WHO	4 (31)	1 (33)	3 (50)	3 (34)	9 (75)	None
**February 1, 2019**						
**Total no. of criteria** **used by all countries**	**20**	**3**	**7**	**10**	**13**	**8**
DRC	6 (30)	1 (33)	1 (14)	4 (40)	6 (46)	2 (25)
Rwanda	6 (30)	1 (33)	1 (14)	5 (50)	None	2 (25)
South Sudan	10 (50)	None	4 (57)	3 (30)	None	None
Uganda	6 (30)	None	2 (26)	4 (40)	7 (54)	5 (63)
WHO	4 (20)	1 (33)	3 (43)	3 (30)	9 (69)	None

**Abbreviation:** POE = point of entry (a border crossing where travelers were screened for Ebola).

* “None” indicates that the country or WHO had no criteria for this category.

Most countries listed nonbleeding symptoms of Ebola as criteria in suspected and community alert categories, except Uganda, where Ebola symptom criteria were limited to signs of unusual bleeding (Table 2). Some definitions had no thresholds for fever, and some had two thresholds concurrently in operation; where thresholds were defined, they ranged from ≥100°F (≥37.8°C) to 101.3°F (38.5°C). By the end of the 6 months, the suspected category included a higher proportion of fever-dependent criteria (65%) than did the community alert category (30%). The proportion of fever-dependent criteria for the community alert category declined from 56% at the start of the outbreak to 30% 6 months later. Except for the case contact and confirmed categories, all countries included more criteria per category than did the WHO definitions. From August 2018 to February 2019, the total number of criteria in the suspected category increased from 12 to 20. Concurrently, concordance decreased from 18% to 5% (Figure). The most consistent criteria for a suspected case among all countries were fever, unexplained bleeding, sudden death, and prior contact with a person with suspected, probable, or confirmed Ebola. However, fever was the only criterion consistently standing alone or upon which other criteria depended.

**TABLE 2:** Ebola virus disease (Ebola) case definition criteria for five case definition categories and fever thresholds during the first 6 months of the 10th Ebola outbreak in the Democratic Republic of the Congo (DRC), with the World Health Organization (WHO) standard Ebola case definition for reference — DRC, Rwanda, South Sudan, and Uganda, August 2018–February 2019

Category/Criteria	DRC	Rwanda	South Sudan	Uganda	WHO
**Community alert**					
Unresponsive fever	Y	—	Y	Y	Y
Sudden onset fever	Y	—	—	—	—
Bloody diarrhea or bloody urine	Y	—	—	Y	—
Sudden death	Y	—	Y	Y	—
Unexplained bleeding	Y	—	Y	Y	Y
Sudden unexplained death and a persistent fever and unexplained bleeding	—	—	Y	—	—
Sudden death in the community of a person who had a strange illness	—	—	Y	—	—
Fever and international travel in the past 21 days	—	—	Y	—	—
Sudden onset fever and severe illness and unexplained bleeding	—	—	Y	—	—
Sudden death and travel to DRC in the past 21 days	Y	Y	—	—	—
Bleeding in the eyes or urine	Y	—	—	—	—
Fever and travel to an Ebola affected area	—	—	Y	—	—
Sudden and unexplained death	—	—	Y	—	Y
Travel to DRC in the past 21 days	—	Y	—	—	—
Travel to DRC in the past 21 days and fever or bleeding symptoms	—	Y	—	—	—
Fever that does not respond to typical treatments and one or more bleeding symptom at a point of entry	—	—	—	Y	—
Fever at a point of entry	Y	—	—	—	—
Unexplained bleeding at a point of entry with or without a fever	—	—	—	Y	—
Sudden death at a point of entry	—	—	—	Y	—
Fever at a point of entry and an epidemiologic link to a suspected, probable, or confirmed case of Ebola	—	—	—	Y	—
Visibly ill at a point of entry and travel to DRC in the past 21 days	—	Y	—	—	—
Signs of illness or bleeding at a point of entry	Y	—	—	—	—
Consistently high fever at a point of entry	—	—	Y	—	—
Fever at a point of entry and travel to DRC in the past 21 days	Y	Y	Y	Y	—
**Suspected case**					
Unresponsive fever	—	—	—	Y	—
Sudden fever and contact with a person with suspected, probable, or confirmed Ebola	—	—	Y	—	—
Sudden fever and an epidemiologic link to Ebola	—	—	Y	—	—
Alive or dead with fever and contact with a person with suspected, probable, or confirmed Ebola	Y	Y	Y	Y	Y
Alive or dead with a fever and contact with an ill or dead animal	Y	Y	Y	—	Y
Sudden onset fever and exposure to a mine or cave	—	Y	—	—	—
Sudden onset fever and three symptoms of Ebola	Y	—	Y	—	—
Sudden onset fever and one bleeding symptom	—	—	Y	Y	—
Unresponsive fever and one or more bleeding issue, such as a miscarriage	—	—	Y	—	—
Sudden fever and an epidemiologic link to Ebola	—	—	Y	—	—
Fever and travel to DRC and one or more symptoms of Ebola	—	Y	—	—	—
Fever and travel to DRC	—	—	Y	Y	—
Fever and travel to DRC and an epidemiologic link to Ebola	—	Y	—	—	—
Unexplained bleeding	Y	Y	Y	Y	Y
Unexplained bleeding and travel to DRC	—	—	Y	—	—
Unexplained bleeding and an epidemiologic link to Ebola	—	—	Y	—	—
Three or more symptoms of Ebola and DRC travel or an epidemiologic link to Ebola	—	—	Y	—	—
Sudden and unexplained death	Y	Y	Y	Y	Y
Sudden unexplained death and an epidemiologic link to Ebola	—	—	Y	—	—
Spontaneous miscarriage	Y	—	—	—	—
Fever and signs of Ebola in a person from the Ebola-affected area	Y	—	—	—	—
Sudden death after the person had a fever and bleeding symptoms	—	—	Y	—	—
Sudden fever that does not respond to typical treatment for fever and one or more symptoms of Ebola	—	—	Y	—	—
Fever and signs of Ebola in a person from the Ebola-affected area	Y	—	—	—	—
Fever and travel within 21 days to the affected area and contact with a dead or ill animal	—	Y	—	—	—
Sudden unexplained death and travel to DRC in the past 21 days	—	Y	Y	—	—
**Probable case**					
Clinician suspects Ebola	Y	—	Y	—	—
Suspected case in a person who is dead and has an epidemiologic link to Ebola	Y	Y	Y	—	Y
Suspected case in a person who is alive and has an epidemiologic link to Ebola	Y	—	—	—	—
Suspected case in a person who is dead with an epidemiologic link to a confirmed case	Y	Y	—	—	—
**Confirmed case**					
Suspected or probable case of Ebola with an RT-PCR positive result	—	Y	Y	—	—
Suspected or probable case of Ebola with IgM antibodies to Ebola virus	—	Y	Y	—	—
Suspected case of Ebola with an RT-PCR positive result	Y	—	Y	Y	Y
Suspected case of Ebola with IgM antibodies to Ebola virus	Y	—	Y	Y	Y
Suspected case with Ebola virus isolation	—	—	Y	—	Y
Suspected of probable case of Ebola with GeneXpert and RT-PCR positive results	—	Y	—	—	—
Suspected of probable case of Ebola with GeneXpert positive result	—	—	Y	—	—
Suspected case with a positive laboratory result	Y	—	—	—	—
**Case contact**					
Person was in the same household	Y	—	—	Y	Y
Had direct contact	—	—	—	Y	—
Shared the same room or bed	—	—	Y	—	—
Direct contact with a person with Ebola, alive or dead	Y	—	Y	—	Y
Touched body fluids	Y	—	Y	Y	Y
Direct contact with the body of a person with Ebola at a funeral	—	—	—	—	Y
Attended a burial ceremony of a person with suspected or confirmed Ebola	Y	—	—	Y	—
Gave patient care	Y	—	Y	—	—
Touched soiled linen	Y	—	Y	Y	Y
Was breastfed	Y	—	—	Y	Y
Shared transport	Y	—	—	Y	—
Had animal contact	Y	—	—	—	Y
Ate bushmeat	Y	—	—	—	Y
Had a laboratory exposure	Y	—	—	—	Y
**Fever threshold**					
≥100°F (≥37.8°C)	—	—	Y	—	—
>100°F (>37.8°C)	—	—	Y	—	—
≥100.4°F (≥38°C)	—	Y	—	Y	—
>100.4°F (>38°C)	Y	Y	—	—	—
101.3°F (38.5°C)	—	—	Y	—	—
Elevated temperature	Y	—	—	—	—
Sudden onset fever	—	—	Y	—	Y
Sudden onset of a very high fever	—	—	Y	—	—
Persistently high fever	—	—	Y	—	—
Fever that does not respond to treatment for usual causes of fever	Y	—	Y	Y	—

**Abbreviations: **Y = criterion present in category definition; — = criterion not present in category definition; IgM = immunoglobulin M; RT-PCR = reverse transcription–polymerase chain reaction.

**FIGURE:**
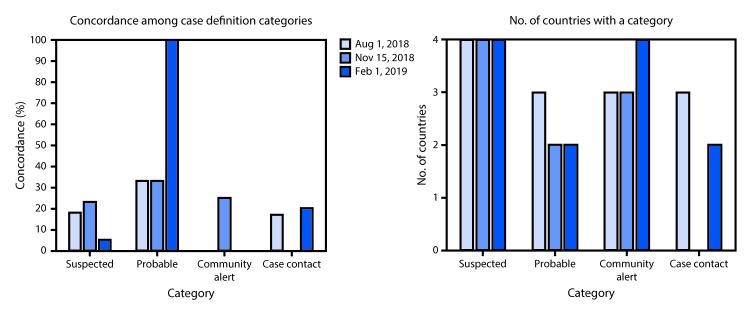
Percentage of concordance of Ebola virus disease category* case definitions and number of countries with case definition categories during the first 6 months of the 10th Ebola outbreak in the Democratic Republic of the Congo — four neighboring countries,^^†^^ August 1, 2018– February 1, 2019 * Not all countries had a case definition for all categories at any or all of the three time points. Concordance is indicated only for the countries that included the category. ^^†^†^ Democratic Republic of the Congo, Rwanda, South Sudan, and Uganda.

The probable category, present at least once for all countries except Uganda, always included the criterion deceased persons with a suspected case with an epidemiologic link to a case. For the DRC case definition, a probable case could be in a person alive with an epidemiologic link, and for DRC (at all three dates) and South Sudan (only at the start of the outbreak) a probable case could be based on a clinician’s suspicion, even without an epidemiologic link. Percentage concordance for the probable category ranged from 33% to 100% (Figure). Laboratory confirmation by RT-PCR or detection of IgM antibody against Ebola virus in a suspected case were typically required for a confirmed case designation (Table 2).

Concordance in the criteria for case contact remained consistently low, increasing from 17% to 20% over the 6-month period (Figure). Initially there was zero concordance for community alerts; concordance increased to 25% in November 2018 but declined back to zero by February 2019. DRC defined point of entry criteria within the community alert category at the start of the outbreak. Rwanda and Uganda added point of entry categories by February 2019; Rwanda’s point of entry criteria were very similar to its criteria for community alert, and Uganda’s were the same. South Sudan briefly included point of entry alert in October 2018 but removed it by February 2019.

## Discussion

Because of the high volume of cross-border population movement between DRC and neighboring countries, strengthening binational and multinational public health communication and coordination is a growing priority. The four contiguous countries are currently reviewing their case definitions and developing procedures to engage in cross-border and regional collaborations that respond to and accommodate differences in case definitions to prevent cross-border transmission of Ebola. Case definitions might not move toward concordance among countries responding to an outbreak and countries in different stages of preparation for possible outbreak spread (*3*,*4*). This analysis found a sustained low level of concordance in Ebola case definitions among DRC and three neighboring countries throughout revisions made over the first 6 months of the outbreak. As the number of criteria increased, case definitions became more complex, and concordance among countries decreased.

DRC is operating in a response phase, and case definitions in the preparedness-phase countries might need to vary in sensitivity thresholds to identify cases based on available resources, perceived level of risk, and competing priorities (*5*). Complexity of and discordance in case definitions affect information sharing about alerts and cases across national borders. The potential risk associated with this discordance to cross-border communication and collaboration during an outbreak with a threat of cross-border spread might warrant a move toward regional harmonization or tailored binational and multinational communication strategies.

The findings in this report are subject to at least two limitations. First, although there was variation among countries in case definition sensitivity, this analysis did not evaluate the effect of discordance on surveillance; cases with in-country transmission have been limited to DRC. Second, MOH-approved case definitions are at the national level and might not represent those used by stakeholders at all levels, where local or cross-border informal information sharing might occur.

Awareness of differences in case definitions across the region provides critical fact-based support to national governments, regional or multinational bodies, and other public health stakeholders as they engage in or shift to preparedness and response initiatives with enhanced cross-border collaboration. Countries should consider routine evaluation of case definitions and implement systematic approaches to harmonization, when possible, and accommodate country-level differences when necessary. Revisiting these strategies throughout the continuum of preparedness and response might reduce the likelihood of cross-border transmission.

**Summary**

**What is already known about this topic?**

Whereas countries might initially base case definitions on global standards, historical context and perceived risk often affect why countries modify and adapt disease case definitions over time, moving either toward or away from regional harmonization.

**What is added by this report?**

Even with a regional risk for Ebola virus disease (Ebola) (i.e., importation into three countries bordering the Democratic Republic of the Congo), Ebola case definitions became increasingly complex and less concordant during a 6-month period.

**What are the implications for public health practice?**

The low level of concordance in case definitions among countries, when case definitions are critical to many outbreak response and preparedness activities, indicates the need for routine evaluation of regional differences in case definitions and implementation of systematic approaches to advance harmonization.

## References

[1] World Health Organization. Ebola virus disease in the Democratic Republic of the Congo—operational readiness and preparedness in neighbouring countries. Disease outbreak news. Geneva, Switzerland: World Health Organization; 2018. https://www.who.int/csr/don/14-august-2018-ebola-drc/en/

[2] World Health Organization. Case definition recommendations for Ebola or Marburg virus diseases. Geneva, Switzerland: World Health Organization; 2014. https://www.who.int/csr/resources/publications/ebola/case-definition/en/

[3] Desclaux A, Malan MS, Egrot M, Sow K, Akindès F; Sow K for EBSEN Study Group, Akindès F for EBO-CI Study Group. Surveillance in the field: over-identification of Ebola suspect cases and its contributing factors in West African at-risk contexts. Glob Public Health 2019;14:709–21. 10.1080/17441692.2018.1534255 30319027

[4] Merrill RD, Rogers K, Ward S, Responding to communicable diseases in internationally mobile populations at points of entry and along porous borders, Nigeria, Benin, and Togo. Emerg Infect Dis 2017;23. 10.3201/eid2313.170520 29155668 PMC5711311

[5] Fitzner J, Qasmieh S, Mounts AW, Revision of clinical case definitions: influenza-like illness and severe acute respiratory infection. Bull World Health Organ 2018;96:122–8. 10.2471/BLT.17.194514 29403115 PMC5791775

